# This Won’t Last Forever: Benefits and Costs of Anticipatory Nostalgia

**DOI:** 10.3389/fpsyg.2020.577308

**Published:** 2020-10-29

**Authors:** Xinyue Zhou, Rong Huang, Krystine Batcho, Weiling Ye

**Affiliations:** ^1^Department of Marketing, Zhejiang University, Hangzhou, China; ^2^Economics and Business Department, Saint Anselm College, Manchester, NH, United States; ^3^Department of Psychology, Le Moyne College, Syracuse, NY, United States; ^4^Department of Marketing, College of Business, Shanghai University of Finance and Economics, Shanghai, China

**Keywords:** anticipatory nostalgia, personal nostalgia, enjoyment, anxiety, affect

## Abstract

What helps consumers extract the greatest happiness from their experiences? The current investigation is the first to introduce to the consumer literature the construct of anticipatory nostalgia, defined as missing aspects of the present before they vanish in the future. While personal nostalgia involves fond memories and longing for what has already been lost, anticipatory nostalgia involves missing what has not yet been lost. In four studies, we show that marketing communications can elicit anticipatory nostalgia, and this emotion can either enhance or reduce consumer enjoyment of the experience, depending on the experience valence or the individual’s level of life satisfaction. Specifically, mediated by anxiety, anticipatory nostalgia decreased enjoyment and positive affect in pleasant situations, but it enhanced enjoyment and affect in unpleasant circumstances. Study 4 extended the paradigm to a real-life setting and showed that the impact of anticipatory nostalgia on enjoyment and meaningfulness can last as long as 8 h after the manipulation.

## Introduction

People frequently feel nostalgic for their past. But can they feel nostalgic for their present? What if people become aware in the moment that the present will be missed 1 day in the future? Knowing that the present will be missed presents a unique opportunity to feel nostalgic, as if the present is experienced as prematurely past. This paper introduces to the consumer literature the construct of anticipatory nostalgia, which is defined as missing aspects of the present before they are gone. Whereas personal nostalgia is a longing for what has already been lost, anticipatory nostalgia involves a longing for what has not yet been lost (Batcho and Shikh, 2016). At one time or another, likely more so in momentous life experiences (e.g., graduations, holidays, anniversaries), people can be struck by feelings of anticipatory nostalgia, as exemplified by the words of singer Trace Adkins, “You may not know it now, but you’re gonna miss this”(Gorley and Miller, 2007). People may also initiate anticipatory nostalgia on tough days (e.g., caring for ill relative, breakup in a relationship) so that they can make the present moment easier to live with. When the whole world was struggling with the 2020 epidemic, we find a blog titled “COVID-19 lockdown nostalgia: It was a scary time, but I will miss our enforced family togetherness” (Dietitian Farida, 2020).

What is the consequence of bringing nostalgia into the present? Will anticipatory nostalgia sweeten the present with the soft glow of reminiscence? Or will it dilute the present enjoyment by considering it already gone? This paper aims to investigate whether anticipatory nostalgia elevates or impoverishes consumer experience. Our findings provide relevant insights into how to enhance consumer experiences. What can companies do to help consumers extract the greatest happiness from an experience? While existing research provides little guidance about how to maximize consumer enjoyment from an experience, companies sometimes use promotion communication that can trigger anticipatory nostalgia. For example, Kodak ads included the messages “life’s little moments don’t stay forever” and “don’t cry over missed special moments.” Disney used the taglines “let the memories begin” and “unforgettable happens here.” An ad about global warming warned “winter, you will miss it when it’s gone.” These marketing communications can elicit anticipatory nostalgia. But how can this affect consumer experience? Will we live better or worse as a result of such messaging?

## Nostalgia and Anticipatory Nostalgia

Although specific definitions of nostalgia vary, personal nostalgia is commonly understood to denote currently missing aspects of one’s lived past (Batcho, 1995, 2013a). Definitions of nostalgia as “a sentimental longing or wistful affection for the past” (Sedikides et al., 2015a, p. 52; Sedikides et al., 2015b, p. 195) and as “missing or longing for something in the past” (Batcho, 1995) share the common element of longing for the past. Definitions that include the component of affection for the past often qualify it with descriptors such as “wistful,” that is, “characterized by melancholy, longing or yearning.” Both theoretical approaches conceptualize personal nostalgia as a blended bittersweet emotion. In prior research based upon the two definitions, personal nostalgia has been associated with a number of psychological benefits, including enhanced consumer patience (Huang et al., 2016), social connectedness (Werman, 1977; Batcho, 1998; Wildschut et al., 2006, 2010; Zhou et al., 2008), social competence and relationship satisfaction (Juhl et al., 2012), continuity of self (Iyer and Jetten, 2011; Sedikides et al., 2015a), identity exploration (Batcho et al., 2008), self-esteem (Vess et al., 2012; Cheung et al., 2013), meaningfulness (Sedikides et al., 2004; Batcho, 2007; Routledge et al., 2008), and social-emotional coping, goal-directed strategies, and positive reframing (Batcho, 2013b). Although nostalgia is believed to be universal, research suggests that its benefits might vary as a function of traits such as neuroticism, neuro-excitability for sadness, and narcissism (Barrett et al., 2010; Köneke, 2010; Hart et al., 2011), as well as habitual worrying (Verplanken, 2012), the belief in identity continuity (Iyer and Jetten, 2011), or social support (Wildschut et al., 2010).

Another recent approach has raised the possibility that the impact of nostalgia might depend upon the time perspective governing the emotion. What if aspects of the present are missed as if they were already past? Recent research has introduced the construct of anticipatory nostalgia, in reference to a tendency to miss elements of the present before they are past by imagining them from a future vantage point (Batcho and Shikh, 2016).

Anticipatory nostalgia, defined as missing the present prematurely before it has become past, has been distinguished from personal nostalgia, which is a psychological state of missing and longing for what has already been lost (Batcho and Shikh, 2016; Batcho, 2020). Although correlated, anticipatory and personal nostalgia were found to constitute separate types of nostalgia. Whereas personal nostalgia was associated with remembering the past, positive affect, and favorable reactions, anticipatory nostalgia was aligned with thinking of the future, emotional distancing, difficulty enjoying the present, and a greater tendency to sadness and worry (Batcho and Shikh, 2016).

By definition, when anticipatory nostalgia is primed, an imagined future may create a conflict between an actual present and a hypothetical future. With the “someday past” still present, anticipatory nostalgia is contingent upon first mentally creating the condition that gives rise to the missing element(s). Cognitively complex, anticipatory nostalgia involves the experience of the present, an imagined future, and an imagined future past (Batcho and Shikh, 2016; Batcho, 2020).

Anticipatory nostalgia should not be confused with anticipated nostalgia. Anticipated nostalgia is the prediction or expectation that one will feel nostalgic for an aspect of the present in the future, not feeling nostalgic in the present (Cheung et al., 2020). Predicting future nostalgia is an interesting cognitive process but not identical to the emotional phenomenon of feeling nostalgic before the future loss occurs. A person can expect to miss a loved one when they leave or die someday, but the expectation doesn’t necessarily include the emotional component of nostalgic missing. Expecting future nostalgia has been shown to predict nostalgia after an important life transition and to be associated with greater savoring of the experience. Post-transition nostalgia was associated with benefits, including enhanced self-esteem, social connectedness, and meaning in life (Cheung et al., 2020). The finding of a greater likelihood of expecting future nostalgia for more positive experiences is not surprising. People would be more likely to expect to miss enjoyable or meaningful events. The line of research by Cheung et al. (2020) reporting positive outcomes of future oriented nostalgia has identified benefits of expecting to be nostalgic in the future, rather than being nostalgic for what has not yet been lost.

## Anticipatory Nostalgia and Consumer Experiences

To date, anticipatory nostalgia has been assessed and explored as a dispositional trait. Research suggests that people prone to anticipatory nostalgia are more likely to experience difficulty enjoying the present and have a greater tendency to worry (Batcho and Shikh, 2016). It is not yet known whether such difficulties characterize anticipatory nostalgia experienced as a passing mood state. Does knowing that the present will be missed 1 day help us appreciate it more deeply or perceive it as more meaningful? Or does it rob the present of pleasure by considering it already gone? Does envisioning its future loss also decrease enjoyment of the present by generating anxiety about an uncertain future?

What impact does an individual’s activation of anticipatory nostalgia have on their current experience? On this issue, existing research has not reached a clear conclusion. Batcho and Shikh (2016) found that positive PANAS intensity was not related to dispositional anticipatory nostalgia; however, negative PANAS intensity was significantly positively related to dispositional anticipatory nostalgia. Meanwhile, regression analyses after adding personal nostalgia traits and other social personality traits found that anticipatory nostalgia predicted sadness and worry. Although it appears that anticipatory nostalgia is more associated with negativity, Batcho and Shikh (2016) also found that people in negative situations (compared to positive situations) show a stronger tendency to view the present from a future perspective, which is characteristic of anticipatory nostalgia, suggesting that anticipatory nostalgia is likely a response to negative situations and that activating anticipatory nostalgia may mitigate negative emotions in a situation. However, the positive or negative effects that activating anticipatory nostalgia can have on an individual’s emotions, and the conditions under which the effects arise, have not been clearly established in previous research.

Batcho and Shikh (2016, p. 78) have made a conjecture based on reasoning that the effect of anticipatory nostalgia may vary depending on the valence of emotional state of the present, but they did not collect empirical evidence to test it. The main basis for their reasoning is that anticipatory nostalgia encompasses both a present experience and a future time perspective, which leads to a conflict between a real present and an imagined future (Batcho and Shikh, 2016). The existence of this temporal perspective difference was consistent with their findings (Batcho and Shikh, 2016). The present study is specifically concerned with whether the consequences of this perspective difference will vary depending on the current contextual valence of the situation.

On the one hand, anticipatory nostalgia may hurt consumer experience, as it entails a conflict between an actual present and an imagined future (Batcho and Shikh, 2016). Imagining an abstract future might generate anxiety associated with uncertainty and worry about the ability to cope with loss and change. Research has shown that consumers who make predictions about uncertain events enjoy observing those events significantly less than those who do not make predictions (Mandel and Nowlis, 2008). Considering uncertain outcomes can decrease enjoyment by producing anticipated regret, and enjoyment is decreased to the extent that outcomes are perceived as more uncertain. Anticipatory nostalgia would be expected to decrease enjoyment by making salient the future uncertainty. Moreover, anticipatory nostalgia may decrease enjoyment by interfering with living fully in the moment. Envisioning the present from a future perspective might prevent one from being mindful of the present and savoring the moment. Mindfulness is a state of bringing one’s attention to experiences occurring in the present moment, during which one is fully aware of what is taking place and immersed in the present (Brown and Ryan, 2003). Mindfulness practices are effective in enhancing life satisfaction, decreasing mood disturbance, and reducing stress and anxiety (Barnes et al., 2007; Querstret and Cropley, 2013; Gu et al., 2015). Research has also identified high involvement or total absorption in an activity to be an important determinant of the subjective experience of leisure (Unger and Kernan, 1983). Because being mindful of the present reduces anxiety and enhances experience, anticipatory nostalgia can diminish the pleasure of an experience by reducing mindfulness and provoking anxieties.

On the other hand, anticipatory nostalgia may enhance consumer experience by making consumers feel that the present is fleeting, thus increasing the perceived value of the present. One study showed that college seniors appreciated their college experience more when they were led to see that they had little time (vs. lots of time) left to enjoy it (Kurtz, 2008). Young people report greater happiness for extraordinary experiences than ordinary ones, whereas happiness from ordinary experiences increases as people get older and perceive their future as less extensive. Such findings suggest that happiness is more intense when an experience is perceived as unlikely to reoccur (Bhattacharjee and Mogilner, 2014). Research has also shown that savoring a nostalgic experience by trying to prolong it can increase consumer patience (Huang et al., 2016). Consumers facing uncertainty connected to a positive event can experience greater, longer-lasting positive feelings when they imagine possible favorable outcomes (Lee and Qiu, 2009). Building on this line of research, we predict that anticipatory nostalgia may enhance experiences by making them more valuable in the moment.

To summarize, previous research and theories seem to suggest that by bringing the future into the present, anticipatory nostalgia can either enhance or diminish consumer experiences of the present. Drawing together these and other diverse lines of evidence showing that both types of effects are possible, we consider the determinants and postulate that the effect of anticipatory nostalgia on consumer experience is dependent on a contextual factor: experience valence. On the one hand, bringing the future into a positive present may hurt the experience. To enjoy a positive experience to its fullest, one needs to be immersed in it and pay full attention to it. Thus, during a positive experience, thinking of the imminent future that is full of uncertainties will provoke anxiety, which will in turn reduce enjoyment of the experience. However, if the experience is negative, shifting attention to the future might remind people that the present is valuable in spite of its difficulties and allow for benefits such as appreciation and reappraisal of the present. Cognitive reappraisal is a positive coping strategy that can reduce stress and anxiety during negative events (Kivity et al., 2016). Therefore, bringing the future into the negative present can prevent ruminating on negative aspects and reduce anxiety, which in turn enhances consumer experience. We put forth the following set of hypotheses:

H1.1. Anticipatory nostalgia reduces consumer enjoyment during a positive experience.
H1.2. During a positive experience, anticipatory nostalgia’s negative influence on the consumer enjoyment is mediated by anxiety.

H2.1. Anticipatory nostalgia enhances consumer enjoyment during a negative experience.
H2.2. During a negative experience, anticipatory’s positive influence on the consumer enjoyment is mediated by anxiety.

## The Present Research

We had three particular aims with the current research. First, we aimed to test whether anticipatory nostalgia can reduce people’s enjoyment of an experience (H1.1). Our second aim was to examine the potential for anticipatory nostalgia to harm the enjoyment of an experience, depending on the valence of experience (H1.1 and H2.1). Our third aim was to examine the possible mechanism of anticipatory nostalgia’s influence on enjoyment of an experience (H1.2 and H2.2).

In order to identify the benefits or disadvantages of anticipatory nostalgia, an assessment method is needed to launch empirical investigations of its functions and correlates. Although the Survey of Anticipatory Nostalgia has shown promise as a measure, it serves to assess dispositional anticipatory nostalgia (Batcho and Shikh, 2016). The present research introduces a transient state measure of anticipatory nostalgia to examine dynamics underlying the impact of premature nostalgia for the present before it becomes the past. The pilot study verified the internal reliability of the measurement of transient state measure of anticipatory nostalgia and distinguished it from the measurement of state personal nostalgia.

By using the transient state measure for anticipatory nostalgia, 4 studies were conducted in the present research. In study 1 we asked individuals to imagine a trip to Disneyland and manipulated anticipatory nostalgia with an advertising slogan. A sample of 120 university students participated in an imaginary trip to Disneyland. We examined the effect of anticipatory nostalgia on consumer enjoyment (H1.1). In study 2 we asked participants to imagine celebrating their birthday, and anticipatory nostalgia was manipulated with a camera advertisement. We studied the effect of nostalgia on consumer enjoyment, and the possible mechanism underlying the effect (H1.1 and H1.2). In study 3 we introduced experience valence as a moderating variable to look at how diverging effects of anticipatory nostalgia can emerge in experiences with different valence (H1.1, H1.2, H2.1, and H2.2). Study 4 extended the paradigm to a real-life setting and assessed the impact of anticipatory nostalgia on enjoyment and meaningfulness 8 h after the manipulation as a function of life satisfaction.

## Study 1

Study 1 manipulated state anticipatory nostalgia to investigate its impact on enjoyment and general affect. Companies selling an experience (such as Disneyland) sometimes use promotional communications that can trigger anticipatory nostalgia to enhance consumer experience. However, according to present research, this kind of advertisement may inadvertently reduce consumer enjoyment. Study 1 tested our hypothesis that activating anticipatory nostalgia may reduce enjoyment. We examined the unintended effect of such communication on consumer enjoyment, as well as positive and negative affect.

### Materials and Methods

#### Participants

Participants were 120 students at a large public university ranging in age from 18 to 32 years (51.7% female; *M*_age_ = 22.2, *SD*_age_ = 2.34). Students received a small monetary stipend for their participation. They were randomly assigned to either the anticipatory-nostalgia condition or the control condition.

#### Procedure and Materials

##### Anticipatory nostalgia

A four-item self-report measure of state anticipatory nostalgia was constructed, guided by the content in the dispositional Survey of Anticipatory Nostalgia (Batcho and Shikh, 2016). The items focused on participants’ nostalgia for anticipated future loss of aspects of the present: “Right now, I know that the present will be missed some day,” “In the future, I will get nostalgic about aspects of my life now,” “In the future, I will often think about things that are happening right now,” and “I expect that I will miss my current lifestyle.” To ensure that the items assessed anticipatory nostalgia as a state, respondents were instructed: “Please rate the following items based on how you feel right now.” Agreement with each of the four statements was indicated on a 7-point scale (1 = “strongly disagree,” 7 = “strongly agree”).

To confirm the reliability of the measure of anticipatory nostalgia and its relationship with personal nostalgia, we recruited a sample of 100 United States participants through Amazon’s Mechanical Turk (MTurk) marketplace for the payment of 0.5 USD. Three respondents failed the attention-check task, resulting in a final sample of 97 adults ranging in age from 20 to 70 years (*M*_age_ = 36.53, *SD*_age_ = 10.55, 60% male). Besides the state measures of anticipatory nostalgia, respondents also completed state measures of personal nostalgia (Wildschut et al., 2006) and self-continuity (Sedikides et al., 2015a), and dispositional measures of construal level (Vallacher and Wegner, 1987), self-esteem (Rosenberg, 1965), sense of power, arousal seeking (Goukens et al., 2009), and socioeconomic status (SES).

The descriptive statistics for the four anticipatory nostalgia items are displayed in Table 1. Principal components factor analysis of this scale yielded a one-factor solution (based on examination of the scree plot as well as Kaiser’s rule that only factors with eigenvalues greater than 1 are extracted). The eigenvalue for factor 1 was 3.16. This single unobserved factor accounted for 79.02% of the variance in the four items. The four items were averaged to form a composite measure to delineate the sample characteristic confounding effects (Wainer, 1976; Dawes, 1979). The correlation between items combined using factor weights and items combined using unit weights was *r* = 1.0. The coefficient for the composite measure was 0.91. One-factor solutions were obtained for female and male participants.

**TABLE 1:** Inter-item correlation, means, and SDs of measures of anticipatory nostalgia.

Variables	1	2	3	4	Corrected item-total correlation
1. Right now, I know that the present will be missed some day	–				0.90
2. In the future, I will get nostalgic about aspects of my life now	0.79**	–			0.92
3. In the future, I will often think about things that are happening right now	0.73**	0.76**	–		0.88
4. I expect that I will miss my current lifestyle	0.69**	0.72**	0.64**	–	0.86
*M*	6.19	6.11	5.74	5.69	
*SD*	1.20	1.27	1.33	1.40	

**p < 0.01.

Further, principal components factor analysis of anticipatory nostalgia and personal nostalgia scales yielded a two-factor solution (based on examination of the scree plot as well as Kaiser’s rule that only factors with eigenvalues greater than 1 are extracted). The eigenvalue for factor 1 was 4.26, and for factor 2 the eigenvalue was 1.76. The first unobserved factor accounted for 60.88% of the variance, and the second factor accounted for 25.19% of the variance. The rotated component matrix demonstrated that anticipatory nostalgia loaded on the first factor, and personal nostalgia loaded on the second factor (Table 2). This analysis showed that the state measure of anticipatory nostalgia was a construct distinct from personal nostalgia.

**TABLE 2:** Rotated component matrix^*a*^ of anticipatory nostalgia and nostalgia measures.

	Component	
	
	Factor 1	Factor 2
Anticipatory Nostalgia: Right now, I know that the present will be missed some day	0.902	
Anticipatory Nostalgia: In the future, I will get nostalgic about aspects of my life now	0.892	
Anticipatory Nostalgia: In the future, I will often think about things that are happening right now	0.819	
Anticipatory Nostalgia: I expect that I will miss my current lifestyle	0.852	
Nostalgia: Right now, I am feeling quite nostalgic (for the past)		0.958
Nostalgia: Right now, I am having nostalgic thoughts (for the past)		0.959
Nostalgia: I feel nostalgic (for the past) at the moment		0.936

*Extraction method: Principal component analysis. Rotation method: Varimax with Kaiser normalization.*

*a. Rotation converged in 3 iterations.*

Consistent with existing research on personal nostalgia, we found that anticipatory nostalgia was not correlated significantly with gender or SES (Anderson et al., 2012b). As expected, the new measure of anticipatory nostalgia correlated positively with measures of personal nostalgia and self-continuity (Table 3). Also consistent with predictions, our results showed that self-esteem, arousal seeking, and sense of power were not correlated with anticipatory nostalgia. Contextual effects could account for the absence of a correlation between the transient measure of anticipatory nostalgia and the dispositional measure of construal level. In general, anticipation might encourage more abstract construal (Vallacher and Wegner, 1987), resulting in greater psychological distance from the present. However, construal level might depend upon context, with abstract construal more attractive in pleasant situations than in unpleasant ones (Labroo and Patrick, 2009). In summary, the four-item, self-report anticipatory nostalgia survey proved to be a coherent, internally consistent measure.

**TABLE 3:** Descriptive statistics and correlations of measures.

Variables	1	2	3	4	5	6	7	8	9	10
1. Anticipatory nostalgia	–									
2. Nostalgia (Wildschut et al., 2006)	0.42**	–								
3. Self-continuity Index (Sedikides et al., 2015a)	0.59**	0.43**	–							
4. Arousal-seeking Tendency (Goukens et al., 2009)	0.08	0.08	0.10	–						
5. Sense of power (Anderson et al., 2012a)	0.09	–0.15	0.04	0.18	–					
6. Self-esteem (Rosenberg, 1965)	0.13	–0.15	0.11	0.04	0.53**	–				
7. Construal level (Vallacher and Wegner, 1987)	0.16	0.02	0.17	0.11	0.17	0.19	–			
SES	0.03	–0.05	–0.10	0.25*	0.44**	0.26*	0.13	–		
Age	–0.01	–0.13	0.10	−0.29**	–0.06	0.20	0.09	–0.12	–	
Gender	0.03	0.03	0.02	–0.06	0.18	–0.00	0.11	0.04	0.15	–
*M*	5.92	4.24	5.60	2.42	5.41	4.72	1.60	4.93	36.53	__

**Correlation is significant at the 0.01 level (2-tailed). *Correlation is significant at the 0.05 level (2-tailed).

##### Procedure

Study 1 examined whether marketing communications promoting an experience can elicit anticipatory nostalgia, and we tested the effect of such communication on consumer enjoyment, as stated in hypothesis 1.

All participants were instructed to imagine that they went to Disneyland for a weekend. They were asked to imagine the specific activities they were doing there and how much fun they were having. Participants worked at their own pace, with no time constraints. Following the imagination exercise, participants were presented with one of two versions of an advertisement for Walt Disney World Resort Annual Passholder webpage (see figure on https://thedisneyblog.com/2011/09/15/disney-world-annual-pass-advertisement/). The original slogan on the advertisement was removed, and participants in the anticipatory-nostalgia condition saw an advertisement with the slogan “You will miss this moment when it’s gone.” Those in the control condition saw the same advertisement without the slogan. Participants imagined that they saw this advertisement when they were in Disneyland. They were asked to evaluate the ad for attractiveness and beauty on a 7-point scale (1 = “not at all,” 7 = “very much”).

Next, participants were asked to evaluate their imaginary Disneyland experience by rating how much they enjoyed themselves, how happy they were, how much fun they had, and how involved they were on a 7-point scale (1 = “not at all” 7 = “very much”). These items were adapted from previous research measures of consumer enjoyment (Nowlis et al., 2004; Mandel and Nowlis, 2008; Miller et al., 2008). The average ratings for the four items comprised a composite score of enjoyment (α = 0.86). Participants then completed a measure of their current affect, the Positive and Negative Affect Schedule (PANAS; Watson et al., 1988). The PANAS consists of 10 items assessing positive affect (PA) with descriptors such as “interested” and “enthusiastic” (α = 0.92) and 10 items assessing negative affect (NA) with descriptors such as “distressed” and “upset” (α = 0.88). Items were rated on a 5-point scale (1 = “very slightly or not at all,” 5 = “extremely”).

Finally, participants completed the transient state anticipatory nostalgia measure developed in the pilot study, with responses averaged to yield a composite index of state anticipatory nostalgia (α = 0.91), followed by the three manipulation-check items for state personal nostalgia (Wildschut et al., 2006). Responses to the three items were averaged to provide an index of personal nostalgia (α = 0.83). Participants also reported their age and gender.

### Results

#### Manipulation Checks

To confirm that the manipulation of anticipatory nostalgia was successful, average anticipatory-nostalgia scores were analyzed in an independent *t*-test. Confirming that the anticipatory-nostalgia manipulation was effective, participants in the anticipatory-nostalgia condition reported feeling more anticipatory nostalgia (*M* = 4.90, *SD* = 1.41) than did their counterparts in the control condition [*M* = 3.95, *SD* = 1.68; *t*(118) = −3.37, *p* = 0.001, *d* = 0.61].

To confirm that the anticipatory-nostalgia manipulation did not influence the level of personal nostalgia, participants’ self-reported nostalgia scores were analyzed in an independent *t*-test. As expected, personal-nostalgia scores for the anticipatory condition (*M* = 5.28, *SD* = 1.02) did not differ from those for the control condition [*M* = 5.41, *SD* = 1.25; *t*(118) = 0.64, *p* = 0.522, *d* = 0.12], suggesting that the manipulation did not affect personal nostalgia. Meanwhile, the correlation coefficient of anticipatory and personal nostalgia was non-significant, *r*(120) = 0.17, *p* = 0.060.

Ratings of attractiveness, *t*(118) = 0.55, *p* = 0.582, *d* = 0.097, and beauty, *t*(118) = 0.68, *p* = 0.499, *d* = 0.12, of the advertisement did not differ between the two groups, suggesting that ad attractiveness was not the driving force behind the main findings.

#### Enjoyment

The average ratings of enjoyment (α = 0.86) were analyzed in an independent *t*-test. Consistent with our prediction, participants in the anticipatory-nostalgia condition felt less enjoyment (*M* = 3.84, *SD* = 1.52) than did those in the control condition [*M* = 4.64, *SD* = 1.52; *t*(118) = 2.89, *p* = 0.005, *d* = 0.53]. Exposure to an advertisement that elicited anticipatory nostalgia reduced participants’ enjoyment of the experience. Consistent with this result, participants’ enjoyment level was negatively correlated with their anticipatory-nostalgia score [*r* (120) = −0.248, *p* = 0.006]. The more anticipatory nostalgia participants felt, the less enjoyment they tended to have.

#### Positive and Negative Affect

The researchers further examined positive (α = 0.92) and negative (α = 0.88) emotions on the PANAS scale. The results were consistent with the hypothesis of a decrease in enjoyment, with less positive emotions (*M* = 2.89, *SD* = 1.02) expected in the anticipatory nostalgic group than in the control group [*M* = 3.42, *SD* = 0.92; *t*(118) = 3.03, *p* = 0.003, Cohen’s *d* = 0.55], and higher negative emotions in the nostalgia group (*M* = 1.53, *SD* = 0.59) than those in the control group [*M* = 1.23, *SD* = 0.44; *t*(118) = −3.10, *p* = 0.002, Cohen‘s *d* = 0.57]. To conclude, the subjects who were primed for anticipatory nostalgia (compared to the control group) in the positive situation felt less positive emotions and more negative emotions. The results of the PANAS analysis were consistent with the findings on enjoyment.

### Discussion

Contrary to theories that posit a favorable impact of anticipation (Mandel and Nowlis, 2008), exposure to an advertisement with an anticipatory-nostalgia slogan intended to enhance consumers’ experience decreased enjoyment and general positive affect. Consistent with hypothesis 1.1, the present results support the unfavorable impact of anticipated nostalgia. However, study 1 leaves several questions unanswered. What force drives the reduced enjoyment and positive affect? Can the result be generalized to other triggers of anticipatory nostalgia and other contexts? Study 2 addresses these questions.

## Study 2

In study 1, anticipatory nostalgia reduced enjoyment and general positive affect. The objective of study 2 was to examine the underlying mechanism of this effect (H1.2). Existing research on dispositional anticipatory nostalgia suggests that anticipatory nostalgia arouses anxiety, which is provoked by bringing an uncertain future into the present. Study 2 examined whether elevated anxiety reduces enjoyment and positive affect. Also, to determine whether the impact of anticipatory nostalgia generalizes beyond the specific task used in the first study, study 2 employed a different scenario and a different manipulation of anticipatory nostalgia.

### Materials and Methods

#### Participants

Participants were 120 students at a large public university who volunteered in exchange for a small monetary payment (63.3% female; *M*_age_ = 22.3, *SD*_age_ = 2.69). They were randomly assigned to either the anticipatory-nostalgia condition or the control condition.

#### Procedure and Materials

All participants first were asked to imagine that it was their birthday and that they were having a party to celebrate. They were asked to imagine how happy they were at the party, and in detail the specific activities they were engaged in at the party. Participants worked at their own pace, with no time constraints. Participants in the anticipatory-nostalgia condition were then asked to imagine that they saw a camera advertisement on TV with the message, “capture this moment, because you will miss it soon.” They were instructed to imagine the feelings this advertisement stirred in them. Those in the control condition were prompted to imagine that they saw a camera advertisement and were asked to relate the feelings this ad stirred in them.

All participants then rated how much they would enjoy their birthday party on the same four items used in study 1. Participants also rated their current emotions on the PANAS as in study 1. Next, participants rated their anxiety level on four items (Marteau and Bekker, 1992): “right now I feel anxious,” “I feel nervous,” “I am nervous,” and “right now I cannot calm myself down.” Participants indicated their level of agreement with each of these four statements on a 7-point scale (1 = “strongly disagree,” 7 = “strongly agree”). Ratings were averaged across the four items to serve as a composite score of anxiety (α = 0.71). Finally, participants completed the manipulation-check items for transient state anticipatory and personal nostalgia as in study 1 and reported their gender and age.

### Results

#### Manipulation Checks

As in study 1, a composite measure of transient state anticipatory nostalgia was obtained by averaging the four items (α = 0.84). Confirming the effectiveness of the anticipatory-nostalgia manipulation, an independent *t*-test indicated that participants in the anticipatory-nostalgia condition felt more anticipatory nostalgia (*M* = 4.63, *SD* = 1.70) than did those in the control condition [*M* = 3.88, *SD* = 1.46; *t*(118) = 2.63, *p* = 0.010, *d* = 0.48]. Moreover, the anticipatory-nostalgia manipulation did not influence the level of personal nostalgia [*M*_anticipatory nostalgia_ = 5.34, *SD*_anticipatory nostalgia_ = 1.23; *M*_control_ = 5.19, *SD*_control_ = 1.43; *t*(118) = 0.62, *p* = 0.539, *d* = 0.11], even though personal and anticipatory nostalgia were positively correlated, *r* = 0.24, *p* = 0.007.

#### Enjoyment

As in study 1, we averaged the four ratings of enjoyment (α = 0.78). Consistent with our prediction, an independent *t*-test revealed that participants in the anticipatory-nostalgia condition felt less enjoyment (*M* = 3.65, *SD* = 1.36) than did those in the control condition [*M* = 4.23, *SD* = 1.35, *t*(118) = −2.33, *p* = 0.022, *d* = 0.43]. Again, we found that the anticipatory-nostalgia manipulation reduced participants’ enjoyment. Furthermore, participants’ enjoyment level was negatively correlated with their anticipatory-nostalgia score [*r* (120) = −0.313, *p* < 0.001]. The more anticipatory nostalgia participants felt, the less enjoyment they tended to have. Hypothesis 1.1 was validated again.

#### Positive and Negative Affect

The researchers further examined positive (α = 0.92) and negative (α = 0.88) emotions on the PANAS scale. Participants in the anticipatory-nostalgia condition felt lower levels of positive affect (*M* = 2.40, *SD* = 0.86) than did those in the control condition [*M* = 3.13, *SD* = 0.96; *t*(118) = −4.42, *p* < 0.001, *d* = 0.81], and higher levels of negative affect (*M* = 1.54, *SD* = 0.51) than did those in the control condition [*M* = 1.32, *SD* = 0.54; *t*(118) = 2.32, *p* = 0.022, Cohen’s *d* = 0.42]. These results were consistent with those in study 1, and with the findings on enjoyment.

#### Mediation Analyses

**FIGURE 1:**
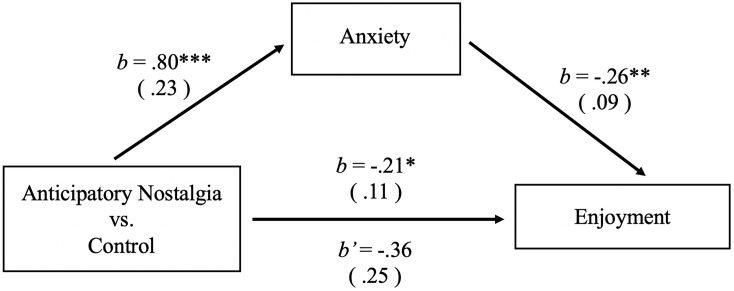
Mediational model tested in study 2. Anxiety partially mediated the effect of anticipatory nostalgia on enjoyment. We found that anticipatory nostalgia enhanced anxiety, which in turn significantly decreased general positive affect (*p < 0.05, **p < 0.01, ***p < 0.001).

To test whether anxiety (α = 0.71) mediated the effects of anticipatory nostalgia on enjoyment, we conducted a simple mediation analysis using 5,000 bias-corrected bootstrap samples with Model 4 of the PROCESS plug-in (Zhao et al., 2010; Hayes, 2013). The results revealed a significant positive effect of anticipatory nostalgia (manipulation condition variable, control = 0, anticipatory nostalgia = 1) on anxiety (*b* = 0.80, *SE* = 0.23, *p* < 0.001, 95% *CI* [0.33; 1.27]) and a significant negative effect of anxiety on enjoyment (*b* = −0.26, *SE* = 0.09, *p* = 0.006, 95% *CI* [−0.45, −0.08]). When anxiety was simultaneously examined as a potential mediator for the effect of anticipatory nostalgia on enjoyment, anticipatory nostalgia was no longer significant (*b* = −0.36, *SE* = 0.25, *p* = 0.150, 95% *CI* [−0.86, 0.13]). More importantly, the indirect effect of anticipatory nostalgia on enjoyment via anxiety was significant (*b* = −0.21, *SE* = 0.11, 95% *CI* [−0.51, −0.06]). Thus, we found that anticipatory nostalgia enhanced anxiety, which in turn significantly decreased enjoyment. Consistent with our prediction, anxiety completely mediated the effect of anticipatory nostalgia on enjoyment (see Figure 1). Thus, Hypothesis 1.2 was validated.

### Discussion

Study 2 makes two contributions, the first of which concerns clarification of the mechanism underlying the effect of anticipatory nostalgia. Although companies readily appeal to anticipatory nostalgia in ads intended to enhance consumer experience, they inadvertently can make consumers more anxious, undermining consumer enjoyment and general positive affect. Second, study 2 replicated and extended study 1’s findings employing a different manipulation of anticipatory nostalgia and utilizing a different type of experience. Study 2 showed that the effect of anticipatory nostalgia is not merely the by-product of a given manipulation or a given experience.

## Study 3

Study 2 showed that anticipatory nostalgia can make people feel anxious, which in turn can lead to less enjoyment of the present moment and less positive emotion. However, the experiences described in studies 1 and 2 were both positive in nature. Whether anticipatory nostalgia plays a different role in negative experiences remains unknown. Existing research suggests that anticipatory nostalgia has a different effect in unfavorable circumstances. If anticipatory nostalgia distances a person from his or her present experiences, participants in an unpleasant situation may feel less anxious and less negative emotion as well as more positive emotion. In study 3, we examined all 4 hypotheses.

### Materials and Methods

#### Participants

We recruited 200 students (78 men and 122 women) from a major public university to participate in the experiment in exchange for a small monetary stipend; they ranged in age from 18 to 39 years (*M*_age_ = 22.6, *SD*_age_ = 3.80).

#### Procedure and Materials

Anticipatory nostalgia and affective valence of the experience were manipulated in a 2 (anticipatory nostalgia or control) × 2 (positive or negative experience) between-subjects design. All participants were asked to imagine they are in Disneyland. Similar to study 1, those in the positive-experience condition were asked to imagine they are having a great time and to visualize in detail what kind of activities they are doing and who they are with. Those in the negative-experience condition were asked to imagine they are having a terrible time in Disneyland. The weather is lousy and the lines are long. They were also asked to imagine in detail what kind of activities they are doing and who they are with. Participants worked at their own pace, with no time constraints.

Participants were then presented with one of the two ads used in study 1 to manipulate their sense of anticipatory nostalgia. They were asked to evaluate the ad for attractiveness and beauty on a 7-point scale (1 = “not at all,” 7 = “very much”). Participants next rated their enjoyment and their emotions on the PANAS as in studies 1 and 2, followed by rating their anxiety level according to the four items used in study 2. Finally, participants completed the manipulation-check items for anticipatory and personal nostalgia and reported their gender and age.

### Results

#### Manipulation Checks

Composite ratings of anticipatory nostalgia averaged over the four items (α = 0.89) were analyzed in a 2 (anticipatory nostalgia or control) × 2 (positive or negative experience) between-subjects design. A significant main effect of the anticipatory-nostalgia manipulation confirmed that participants in the anticipatory-nostalgia condition felt more anticipatory nostalgia (*M* = 4.72, *SD* = 1.53) than did those in the control condition [*M* = 4.02, *SD* = 1.62; *F*(1,196) = 5.74, *p* = 0.002, η^2^ = 0.049]. There was a significant main effect of experience valence [*F*(1,196) = 16.41, *p* = 0.018, partial η^2^ = 0.028]. Respondents in the positive experience group (*M*_positive experience_ = 4.64, *SD* = 1.54) reported more anticipatory nostalgia than those in the negative experience group (*M*_negative experience_ = 4.11, *SD* = 1.65). However, the interaction effect of the two factors on anticipatory nostalgia was non-significant [*F*(1,196) = 0.00, *p* = 0.964, partial η^2^ < 0.001].

Moreover, as in studies 1 and 2, the anticipatory-nostalgia manipulation did not influence the level of personal nostalgia [*M*_anticipatory nostalgia_ = 5.15, *SD*_anticipatory nostalgia_ = 1.38; *M*_control_ = 5.03, *SD*_control_ = 1.33; *F*(1,196) = 0.44, *p* = 0.510, η^2^ = 0.002], even though personal and anticipatory nostalgia were positively correlated, *r* = 0.24, *p* = 0.001. Neither the main effect of experience valence [*F*(1,196) = 1.84, *p* = 0.177, η^2^ = 0.009] nor the interaction [*F*(1,196) = 0.06, *p* = *0.808*, η^2^ < 0.001] was significant.

Meanwhile, neither the attractiveness of the advertisement, *F*(1,196) = 0.20, *p* = 0.652, η^2^ = 0.001, nor ratings of its beauty, *F*(1,196) = 1.85, *p* = 0.175, η^2^ = 0.009, differed between the anticipatory nostalgia and control group, suggesting that the impact of anticipatory nostalgia on enjoyment and affect was not driven by aesthetic differences between the ads.

#### Enjoyment

A 2 (anticipatory nostalgia or control) × 2 (positive or negative experience) ANOVA on ratings of enjoyment revealed a significant main effect of experience valence on enjoyment, *F*(1,196) = 13.39, *p* < 0.001, η^2^ = 0.06. Participants in the positive-experience group indicated more enjoyment (*M*_positive_ = 4.46, *SD*_positive_ = 1.48) than did those in the negative group (*M*_negative_ = 3.68, *SD*_negative_ = 1.58). The main effect of the anticipatory-nostalgia manipulation on enjoyment was not significant, *F*(1,196) = 0.06, *p* = 0.815, η^2^ < 0.01 (*M*_anticipatory nostalgia_ = 4.10, *SD*_anticipatory nostalgia_ = 1.51; *M*_control_ = 4.05, *SD*_control_ = 1.65).

More interesting, however, was the significant interaction of the two factors, *F*(1,196) = 10.94, *p* = 0.001, η^2^ = 0.05. Confirming our hypothesis, planned comparisons revealed a simple effect of anticipatory nostalgia in the positive- and negative-experience conditions, but in opposite directions. Specifically, in the positive-experience condition, anticipatory nostalgia reduced enjoyment [*M*_anticipatory nostalgia_ = 4.14, *SD*_anticipatory nostalgia_ = 1.48; *M*_control_ = 4.80, *SD*_control_ = 1.45; *F*(1,196) = 4.68, *p* = 0.032, *d* = 0.44]; whereas in the negative-experience condition, anticipatory nostalgia increased enjoyment [*M*_anticipatory nostalgia_ = 4.07, *SD*_anticipatory nostalgia_ = 1.56; *M*_control_ = 3.31, *SD*_control_ = 1.53; *F*(1,196) = 6.31, *p* = 0.013, *d* = 0.49]. Thus, hypotheses 1.1 and 2.1 were validated again.

#### Positive and Negative Affect

Using PANAS positive emotion as the dependent variable, a 2 (anticipatory nostalgia group vs. control group) × 2 (positive vs. negative experience) ANOVA indicated that the manipulation of anticipatory nostalgia reduced positive emotion in the positive situation but elevated positive emotion in the negative situation. There was a significant main effect of experience type [*F*(1,196) = 16.41, *p* < 0.001, partial η^2^ = 0.08]. Respondents in the positive experience group (*M*_positive experience_ = 3.24, *SD* = 0.92) reported more positive emotions than those in the negative experience group (*M*_negative experience_ = 2.75, *SD* = 0.84). The main effect of anticipatory nostalgia on positive emotions was not significant (*M*_anticipatory nostalgia_ = 2.98, *SD* = 0.90; *M*_control_ = 3.01, *SD* = 0.93; *F*(1,196) = 0.04, *p* = 0.849, partial η^2^ < 0.01). A significant interaction effect was expected between nostalgia and experience type [*F*(1,196) = 16.01, *p* < 0.001, partial η2 = 0.08]. Further simple effects tests revealed that, for positive experiences, the anticipatory nostalgia manipulation reduced positive emotions [*M*_anticipatory nostalgia_ = 2.99, *SD* = 0.99; *M*_control_ = 3.49, *SD* = 0.78; *F*(1,196) = 8.79, *p* = 0.003, partial η^2^ = 0.04]; however, for negative experiences, anticipatory nostalgia increased positive emotions [*M*_anticipatory nostalgia_ = 2.98, *SD* = 0.81; *M*_control_ = 2.52, *SD* = 0.82; *F*(1,196) = 7.26, *p* = 0.008, partial η^2^ = 0.04].

Similarly, the negative emotion of PANAS was used as the dependent variable to again verify the findings obtained in study 2. The results of the 2 (anticipatory nostalgia group vs. control group) × 2 (positive vs. negative experience) ANOVA indicated that the manipulation of anticipatory nostalgia increased the negative emotion of the subjects in the positive situation, but decreased the negative emotion of the subjects in the negative situation. There was a significant main effect of experience type [*F*(1,196) = 32.69, *p* < 0.001, partial η^2^ = 0.14], and subjects in the positive experience group (*M*_positive_ = 1.40, *SD*_positive_ = 0.47) reported fewer negative emotions than the negative experience group did (*M*_negative_ = 1.90, *SD*_negative_ = 0.79). The main effect of anticipatory nostalgia on negative emotion was not significant [*M*_anticipatory nostalgia_ = 1.60, *SD* = 0.61; *M*_control_ = 1.70, *SD* = 0.78; *F* (1,196) = 1.33, *p* = 0.250, partial η^2^ = 0.01]. In contrast, there was a significant interaction effect between anticipatory nostalgia and experience type [*F*(1,196) = 21.16, *p* < 0.001, partial η^2^ = 0.10]. Further simple effects tests revealed that, for positive experiences, the anticipatory nostalgia manipulation increased negative emotions [*M*_anticipatory nostalgia_ = 1.55, *SD* = 0.55; *M*_control_ = 1.25, *SD* = 0.32; *F*(1,196) = 5.94, *p* = 0.016, partial η^2^ = 0.03]; for negative experiences, the anticipatory nostalgia manipulation reduced negative emotions [*M*_anticipatory nostalgia_ = 1.65, *SD* = 0.66; *M*_control_ = 2.16, *SD* = 0.83; *F*(1,196) = 16.54, *p* < 0.001, partial η^2^ = 0.08].

These results showed the same pattern as those in study 1 and study 2, and were also consistent with the findings on enjoyment.

#### Anxiety

**FIGURE 2:**
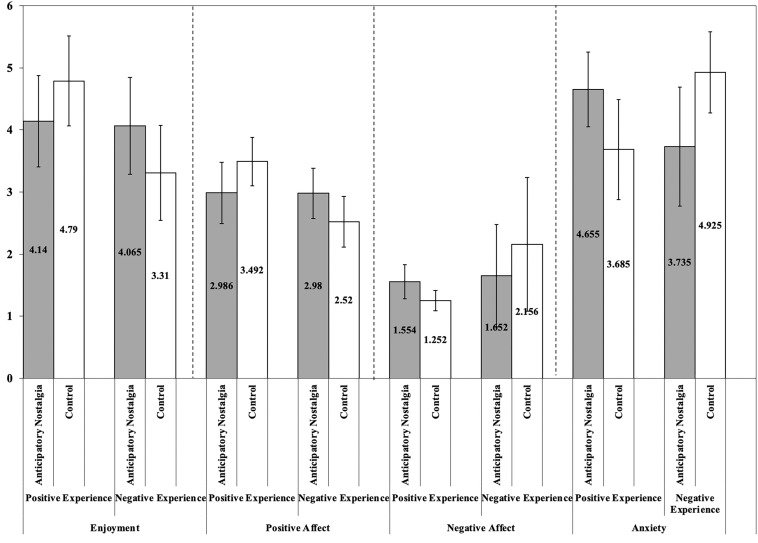
Study 3 enjoyment, positive affect, negative affect and anxiety of participants in 2 (Anticipatory nostalgia vs. Control) × 2 (Positive vs. Negative experience) groups. Anticipatory nostalgia produced directionally different effects for those with positive experiences and those with negative experiences. Specifically, in the positive-experience condition, anticipatory nostalgia increased anxiety, whereas in the negative-experience condition, anticipatory nostalgia reduced anxiety.

A 2 (anticipatory nostalgia or control) × 2 (positive or negative experience) ANOVA on anxiety scores yielded non-significant main effects of anticipatory-nostalgia condition [*F*_anticipatory nostalgia_ (1,196) = 0.26, *p* = *0.613*] and experience valence [*F*_valence_ (1,196) = 0.54, *p* = *0.462*]. Importantly, the interaction between anticipatory nostalgia and experience valence was significant, *F*(1,196) = 24.70, *p* < 0.001, η^2^ = 0.11 (Figure 2). Here again, anticipatory nostalgia produced directionally different effects for those with positive experiences and those with negative experiences. Specifically, in the positive-experience condition, anticipatory nostalgia increased anxiety [*M*_anticipatory nostalgia_ = 4.66, *SD*_anticipatory nostalgia_ = 1.21; *M*_control_ = 3.69, *SD*_control_ = 1.62; *F*(1,196) = 9.96, *p* = 0.002, partial η^2^ = 0.05], whereas in the negative-experience condition, anticipatory nostalgia reduced anxiety [*M*_anticipatory nostalgia_ = 3.74, *SD*_anticipatory nostalgia_ = 1.92; *M*_control_ = 4.92, *SD*_control_ = 1.31; *F*(1,196) = 14.99, *p* < 0.001, partial η^2^ = 0.07].

#### Moderated Mediation

**FIGURE 3:**
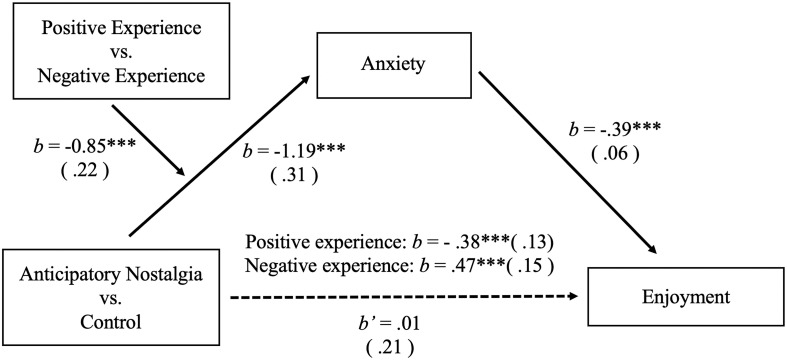
Moderated mediational model tested in study 3. We found significant moderated mediation effects of valence of experience on the mediation effects of anxiety (***p < 0.001).

To investigate the moderated effect of valence of experience on the mediation effect of anxiety on the influence of anticipatory nostalgia (manipulation condition variable, control = 0, anticipatory nostalgia = 1) on enjoyment, a bootstrapped moderated mediation analysis with 5,000 bootstrapped samples was performed (Model 7, Zhao et al., 2010; Hayes, 2013). We found significant moderated mediation effects of valence of experience (manipulation condition variable, negative = 0, positive = 1) on the mediation effects of anxiety (*b* = −0.85, *SE* = 0.21, 95% *CI* [−1.33, −0.46]). In the negative-valence experience condition, the indirect path from anticipatory nostalgia to enjoyment was significantly positive (*b* = 0.47, *SE* = 0.15, 95% *CI* [0.22, 0.81]), whereas in the positive-valence experience condition, the indirect path was significantly negative (*b* = −0.38, *SE* = 0.13, 95% *CI* [−0.67, −0.15]; Figure 3). When anxiety ratings were simultaneously examined as potential mediators for the effect of anticipatory nostalgia on enjoyment, the effect of anticipatory nostalgia was no longer significant (*b* = 0.01, *SE* = 0.21, 95% *CI* [−0.40, 0.41]). Thus, hypotheses 1.2 and 2.2 were validated again.

### Discussion

As in studies 1 and 2, we again found that anticipatory nostalgia reduces enjoyment and general positive affect in the positive-experience condition. However, study 3 revealed that this effect can be reversed when the experience is negative. Follow-up analyses confirmed that this interaction was mediated by anxiety. That is, anticipatory nostalgia increases anxiety in a positive experience, which in turn reduces enjoyment and decreases positive affect. On the other hand, anticipatory nostalgia reduces anxiety in a negative experience, which in turn enhances enjoyment and boosts positive affect.

## Study 4

In the first three studies, we found that anticipatory nostalgia influenced participants’ perceived enjoyment in an imagined scenario task. But can anticipatory nostalgia exert an influence in real-life settings? How long does the effect last? To determine if the moderating effect of anticipatory nostalgia would replicate in real life, study 4 examined its influence in a real-life setting. The second objective of study 4 was to examine the duration of the effect in real life. Specifically, we investigated whether the impact of a simple manipulation of anticipatory nostalgia on enjoyment of the day would be evident after 8 h. The third objective of study 4 was to examine the effect of anticipatory nostalgia on life meaningfulness. Research has revealed a relationship between personal nostalgia and meaningfulness (Sedikides et al., 2004; Batcho, 2007). Construal theory suggests that the temporal distancing resulting from anticipatory nostalgia might allow for a higher level of cognitive processing. Viewing the present as past might encourage reflection and reappraisal, leading to greater meaningfulness of the experience at the time.

In study 3, we found that anticipatory nostalgia reduces enjoyment in positive experiences but enhances enjoyment in negative experiences. Study 4 examines whether the impact of anticipatory nostalgia would depend upon level of life satisfaction. In particular, would considering less satisfactory experiences from a future mental vantage point dull the negative aspects and enhance their enjoyment and meaningfulness?

### Materials and Methods

#### Pretest for Material

We pretested two versions of campus postcards in order to ensure the validity of anticipatory-nostalgia manipulation. In study 4. 108 students (ranging in age from 18 to 23) were recruited online (65 women, 43 men; *M*_age_ = 20.25, *SD*_age_ = 0.86) and randomly assigned to either the anticipatory-nostalgia condition or control condition.

The two groups were both instructed to go through 6 postcards, read the copy on them, and then choose the one they liked best. Participants in the anticipatory-nostalgia condition saw a set of six campus images with the slogan “Someday, you will miss people and things here.” On each, while those in the control condition saw another set of postcards with the same images but a different slogan “On this beautiful campus, you are surrounded by friendly people.” All the six postcards were presented to the participants in random order. They were required to click the button under the postcard they liked the best. Only one postcard could be chosen.

After that, participants completed the manipulation-check items for anticipatory (α = 0.80) and personal nostalgia (α = 0.86), rated their anxiety level (α = 0.94) on the same measure used in study 1, 2, and 3, and reported their gender and age.

Results revealed that the anticipatory-nostalgia manipulation did influence the level of anticipatory nostalgia [*M*_anticipatory nostalgia_ = 5.64, *SD*_anticipatory nostalgia_ = 0.88; *M*_control_ = 5.23, *SD*_control_ = 0.91; *t*(106) = 2.39, *p* = 0.019, *d* = 0.46], and had no effect on personal nostalgia [*M*_anticipatory nostalgia_ = 5.12, *SD*_anticipatory nostalgia_ = 1.22; *M*_control_ = 4.92, *SD*_control_ = 1.11; *t*(106) = 0.95, *p* = 0.344, *d* = 0.18]. Participants’ anxiety did not differ between the two groups [*M*_anticipatory nostalgia_ = 3.93, *SD*_anticipatory nostalgia_ = 1.40; *M*_control_ = 3.49, *SD*_control_ = 1.59; *t*(106) = 1.56, *p* = 0.122, *d* = 0.30]. The pretest confirmed the sufficient manipulation validity of the campus postcards we would use in study 4.

#### Participants

Participants were 302 university students invited to give their opinions in an activity sponsored by the alumni association. Eight hours after the session, at about 22:30 p.m., participants were contacted for follow-up. By the end of the day, we failed to get in touch with 37 of them to participate in the second part of the study, leaving a final sample of 265 participants (53.4% female; *M*_age_ = 20, *SD*_age_ = 1.07). There were no significant differences in gender, *F*(1,300) = 2.31, *p* = 0.271, or age, *F*(1,300) = 1.24, *p* = 0.483, between the people we failed to contact and those in the final sample.

#### Procedure

Participants were recruited on campus around noon (11:45 a.m. to 1:20 p.m.) to participate in an activity to help the university select its future official image. As an indicator of how positive or negative they perceive their present to be, participants were first asked to rate their current life satisfaction by responding to the question “Right now, how much are you satisfied with your present life?” on a 7-point scale (1 = “not at all,” 7 = “very much”). Next, participants were presented with six different images of the campus and asked to vote for the one they liked best. The students were randomly assigned to either an anticipatory-nostalgia condition or a control condition. We manipulated participants’ anticipatory nostalgia with the material pretested. Specifically, for the anticipatory-nostalgia group, the caption read: “Someday, you will miss people and things here.” For the control group, the caption read: “On this beautiful campus, you are surrounded by friendly people.” Participants were asked to evaluate each of the six images carefully and to vote for the one they liked best. After voting, participants were asked to read the caption and evaluate its fluency (“How fluent do you think this sentence is?” 1 = “not at all,” 7 = “very much”). At the end of the first session, participants left their cell phone number in order to be contacted and told which image won the popular vote by the end of the day.

The purpose of the experiment was not to determine the duration of the effect, so we did not schedule multiple surveys after noon. We were more concerned with meeting the requirements of the experiment while not disrupting the normal campus life of the students. We conducted the second round of interviews around 22:30 p.m. for a number of reasons: first, the school’s study rooms and library close at 22:00, and most students had already returned to their dormitories at 22:30, so being interviewed did not interfere with their studies; second, this time represented the end of the day, making it convenient for us to ask the subjects to evaluate their feelings about the day. Thirdly, the interviewer had to contact the interviewees one by one for the 2nd part of survey. It took about an hour to make all the calls, and we wanted to finish the calls before midnight.

Thus, after about 8 h, participants were contacted and asked to indicate the extent to which they enjoyed themselves that day and to what extent they felt they had a meaningful day on a 7-point scale (1 = “not at all,” 7 = “very much”). Participants were then informed of the image election results, and those who had selected the most popular image received a small monetary reward. Finally, they reported their gender and age.

### Results

#### Enjoyment

ANOVA analysis indicated a non-significant main effect of anticipatory nostalgia on enjoyment of the day (*M*_control_ = 4.91, *SD* = 1.19; *M*_anticipatory nostalgia_ = 5.01, *SD* = 1.14; *F*(1,263) = 0.48, *p* = 0.49). Participants’ ratings of their life satisfaction before the anticipatory-nostalgia manipulation were tested as a possible moderator variable. We investigated whether the two interacted to influence enjoyment reported at the end of the day. First, regression analysis utilizing manipulation condition variable (control = 0, anticipatory nostalgia = 1), life satisfaction, and their interaction term showed a main effect of nostalgia manipulation [β = 2.28, *t*(263) = 3.73, *p* < 0.001] on enjoyment of the day, and a main effect of life satisfaction [β = 0.66, *t*(263) = 8.05, *p* < 0.001]. Further, a significant interaction between anticipatory nostalgia and life satisfaction was identified [β = −0.44, *t*(263) = −3.71, *p* < 0.001].

**FIGURE 4:**
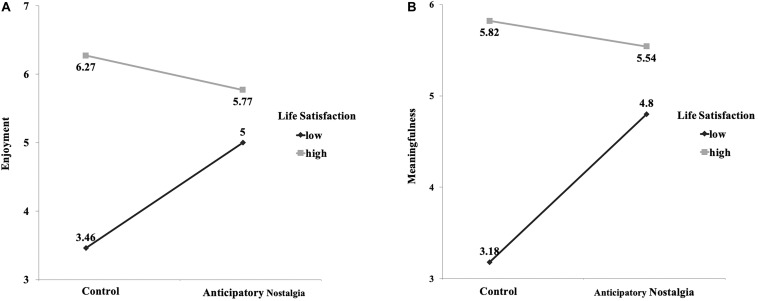
**(A)** Enjoyment of the day of anticipatory nostalgia group and control group, moderated by life satisfaction at noon. For participants with a high life satisfaction (at one standard deviation above the mean: life satisfaction = 6.15), anticipatory nostalgia lowered their enjoyment, whereas the effect was reversed for those with a low life satisfaction. **(B)** Meaningfulness of anticipatory nostalgia group and control group, moderated by life satisfaction at noon. For participants with a higher life satisfaction (at one standard deviation above the average life satisfaction: life satisfaction = 6.15), anticipatory nostalgia did not significantly influence their perceived meaningfulness of the day, whereas anticipatory nostalgia significantly improved the perceived meaningfulness of the day for those with a lower life satisfaction.

Spotlight analyses were utilized to further investigate the moderating effects of life satisfaction (Model 1 in SPSS with 5,000 samples; Hayes, 2013). The model was significant, with a *R*^2^ = 0.47, *p* < 0.001. As expected, we found a significant interaction effect between anticipatory-nostalgia manipulation and earlier life satisfaction [β = −0.44, *t*(260) = −3.71, *p* < 0.001] on enjoyment of the day. For participants with a high life satisfaction (at one standard deviation above the mean: life satisfaction = 6.15), anticipatory nostalgia lowered their enjoyment, β = −0.42, *t*(260) = −2.29, *p* = 0.023, 95% *CI* [−0.78, −0.06], whereas the effect was reversed for those with a low life satisfaction (at one standard deviation below the mean: life satisfaction = 3.90), β = 0.54, *t*(260) = 2.95, *p* = 0.004, 95% *CI* [0.18, 0.90] (Figure 4A). Consistent with the findings from studies 2 and 3, when consumers are satisfied with their lives, anticipatory nostalgia potentially increases anxiety and reduces enjoyment, but when consumers are not satisfied with their lives, anticipatory nostalgia can decrease anxiety and increase enjoyment.

#### Meaningfulness

ANOVA indicated a main effect of anticipatory nostalgia on perceived meaningfulness. In particular, at the end of the day, participants in the anticipatory-nostalgia group felt that they had a meaningful day to a greater extent (*M* = 5.18, *SD* = 1.25) than did those in the control group [*M* = 4.79, *SD* = 1.20; *F*(1,263) = 6.51, *p* = 0.011]. Furthermore, consistent with the impact on enjoyment, anticipatory nostalgia did interact with life satisfaction to influence perceived meaningfulness, β = −0.35, *t*(263) = −2.75, *p* = 0.006. Spotlight analyses were utilized to investigate further the moderating effects of life satisfaction (Model 1 in SPSS with 5,000 samples; Hayes, 2013). The model was significant, with a *R*^2^ = 0.45, *p* < 0.001. As expected, we found a significant interaction effect between the anticipatory-nostalgia manipulation and earlier life satisfaction, β = −0.35, *t*(263) = −2.75, *p* = 0.007, on meaningfulness of the day. For participants with a higher life satisfaction (at one standard deviation above the average life satisfaction: life satisfaction = 6.15), anticipatory nostalgia did not significantly influence their perceived meaningfulness of the day, β = −0.03, *t*(260) = −0.16, *p* = 0.87, 95% *CI* [−0.41, 0.35], whereas anticipatory nostalgia significantly improved the perceived meaningfulness of the day for those with a lower life satisfaction (life satisfaction = 3.98), β = 0.72, *t*(260) = 3.71, *p* = 0.003, 95% *CI* [0.34, 1.10] (Figure 4B). When consumers are not satisfied with their lives, anticipatory nostalgia can increase the perceived meaning of their current experience.

### Discussion

The results of study 4 supported our hypotheses and extended our findings to life beyond the laboratory setting. Anticipatory nostalgia helped individuals who are not fully satisfied with their lives to find greater enjoyment and meaning in their day. However, for those who were already highly satisfied with their lives, anticipatory nostalgia reduced their enjoyment and did not enhance meaningfulness.

## General Discussion

### Conclusion

The studies reported here make an important contribution to the recently conceived area of research on the construct of anticipatory nostalgia. Prior studies measured and explored anticipatory nostalgia as a dispositional trait. The present article examined anticipatory nostalgia as a transient state by introducing a state measure, identified a mechanism underlying the impact of anticipatory nostalgia on enjoyment, documented the effects of contextual valence, and investigated its influence on enjoyment and meaningfulness in a real-life context.

Consistent with available research, the present findings support the usefulness of anticipatory nostalgia as a type of nostalgia, distinct from personal nostalgia. As in prior research with dispositional measures, the present research found a moderate correlation of state measures of anticipatory and personal nostalgia. Overall, the present results add to existing evidence for the conclusion that anticipatory nostalgia is related but not identical to personal nostalgia. Prior research revealed that personal nostalgia predicted a tendency toward happiness, whereas dispositional anticipatory nostalgia was associated with a generalized tendency toward sadness. Similarly, study 2 of the present research found that the manipulation of state anticipatory nostalgia decreased enjoyment and positive affect. Echoing this, singer-songwriter Kris Kristofferson sang about the threat of anticipatory nostalgia to a romantic relationship: “This could be our last goodnight together. We may never pass this way again. Just let me enjoy till it’s over or forever. Please don’t tell me how the story ends” (Kristofferson, 1978).

Importantly, the present research advances the understanding of anticipatory nostalgia by identifying anxiety as one mechanism underlying its impact. Prior research provided evidence that anticipatory nostalgia reflects neither depression nor pessimism but a reluctance to let go of the present. Paradoxically, the desire to hold on to the present might jeopardize full engagement with it. The present studies suggest that anticipating future loss of the present raises anxiety and thereby decreases enjoyment and positive affect. On the other hand, study 3 revealed that when the present is challenging or unpleasant, anticipating change can reduce negative affect by creating temporal distance from the problematic present. Study 4 extended the finding to a real-life application and concluded that anticipatory nostalgia raises anxiety and reduces enjoyment when people are highly satisfied with their lives, but it decreases anxiety and increases enjoyment when people are not fully satisfied with their lives. Construal theory suggests that temporal distance can effect a change in perspective and allow for reappraisal of the present. In a real-life setting, study 4 demonstrated that anticipatory nostalgia enhanced meaningfulness for people who were not fully satisfied with their lives, i.e., it perhaps encouraged a greater appreciation of the moment (for what it is).

The present findings provide insights into how to enhance experiences in a variety of contexts, including marketing, education, and counseling. Marketing strategies that employ methods to trigger anticipatory nostalgia are most effective when they target challenging situations or connect messaging to present annoyance, inconvenience, or difficulty. Marketers can tailor ads, especially on the internet, to address target audiences to arouse anticipatory nostalgia in those who will benefit most from such emotional engagement (caring for an ill relative, coping with the epidemic). Similarly, schools can enhance meaningfulness of their education for students by emphasizing how they’ll miss it someday as well as explaining its future usefulness. Counselors can encourage positive reappraisal by engaging clients in an analysis of present adverse circumstances from an imagined future perspective. Future research is needed to determine the circumstances under which anticipatory nostalgia can be constructive, or when it can be applied therapeutically to provide consolation by reminding people that time is fleeting. The finding in study 4 of enhanced meaningfulness for people not fully satisfied with their lives encourages future research into the possible therapeutic application of anticipatory nostalgia. Applied appropriately, perhaps anticipatory nostalgia can promote adaptive coping. The adoption of a future vantage point provides an opportunity for positive reappraisal of present adversity. Less clear is whether or how such mental distancing can enrich appreciation of the positive aspects of the present and increase attentiveness and engagement by reminding people that the opportunity to enjoy the present will not last forever.

### Limitation and Future Directions

The present studies represent an important contribution to the advancement of research on anticipatory nostalgia. However, the absence of a manipulation check of the experience valence is a shortcoming in study 3. Although the same practice can be found in the existing literature (Nicolao et al., 2009), we recommend that future research measure affect, both positive and negative, immediately after the experience recall task is completed as manipulation check (Zhang et al., 2014). Another limitation is the use of only 100 participants in the factor analysis in the pilot test in study 1. In future research, larger sample size should be used to validate the measures.

Some questions of interest not addressed by these studies suggest opportunities for productive future efforts. Are people more likely to experience anticipatory nostalgia in positive or negative circumstances? Could there be any individual differences leading consumers to respond differently to ads and text that invoke anticipatory nostalgia? Are people prone to anticipatory nostalgia more or less likely to experience the anxiety triggered by anticipatory nostalgia arising in the moment than are less prone individuals? Are individuals with a predisposition to anticipatory nostalgia more or less likely to employ it in unpleasant than in pleasant situations? Is the tendency to employ anticipatory nostalgia associated with age, other personality traits, or is it learned?

Are there additional variables that moderate the influence of anticipatory nostalgia? Future research is needed to determine whether construal level plays a role in mediating the impact of anticipatory nostalgia on enjoyment, positive affect, or meaningfulness. By diminishing involvement in the present, perhaps defensive distancing can threaten the quality of social interactions. It remains for future research to explore whether emotional distancing during unpleasant times ameliorates pain at the price of feeling more alone or lonely. Moreover, studies are worth doing to explore whether would there be any differences between the levels of anticipatory nostalgia invoked by appeals of missing the present in near future (e.g., the next year) vs. distant future (e.g., 30 years later).

By definition, anticipatory nostalgia is a form of nostalgia, so the finding of different effects of anticipatory nostalgia in positive and negative situations raises similar questions about personal nostalgia. Can the benefits of nostalgia for the past also depend upon the context and intervening cognitive variables? It also remains for future research to identify distinctions between dispositional and transient state anticipatory nostalgia. Further research is also needed to distinguish anticipatory nostalgia from other loss related concepts, such as bereavement. By definition, anticipatory nostalgia would be expected to occur when change or loss is expected in the future. A person is not as likely to miss something they don’t expect to be different or lost someday. That said, not all items on the Survey of Anticipatory Nostalgia (Batcho and Shikh, 2016) are characterized by explicit negative tone. Although items such as “someone you love will leave someday” do sample nostalgia for future loss, other items can be interpreted in different ways. For example, an item such as “you’ll move to a new city, home or apartment” can be optimistic and understood as promising better conditions to come (not unlikely for students looking forward to moving on from dormitory living to life after graduation). Similarly, “society will change” can be interpreted positively in terms of scientific and social progress or future resolution of political or other divisions. While some change is accompanied by sadness, much change (especially in contemporary culture focused on scientific and technological progress), represents future improvements. Nostalgia inherently engages positive and negative emotions, which is why questions related to when nostalgia is generally adaptive or maladaptive is of important theoretical and practical interest. Unlike bereavement, anticipatory nostalgia is more likely to involve positive aspects of the future along with the sense of loss. For example, anticipatory nostalgia triggered by the realization that one’s child will not always remain young involves the possibility of many wonderful components of the future such as that child 1 day falling in love, getting married, having children of their own. Distinguishing anticipatory nostalgia from anticipatory grief merits further research, given that the grief work perspective has dominated the bereavement literature (Bonanno and Kaltman, 1999). Anticipatory grief is a term that describes a process in which an individual confronted with impending loss initiates the grieving process in anticipation of that event. It is assumed to be a positive adaptive response to expected loss. A recent study has suggested that anticipatory nostalgia may serve as a strategy to cope with loss (Batcho, 2020). Future research conducted from the perspective of coping strategy is likely to be productive.

Together with the present findings, future research can enrich our understanding of the complex emotion of nostalgia and its implications for commercial application, psychological well-being, and quality of life.

## Data Availability Statement

The dataset for study 4 is not publicly available because participants were not so instructed.

## Ethics Statement

The studies involving human participants were reviewed and approved by the Shanghai University of Finance and Economics College of Business Research Ethics Committee. The patients/participants provided their written informed consent to participate in this study.

## Author Contributions

XZ formulated the research question. XZ, RH, KB, and WY designed the experiments and analyzed the data. XZ, KB, and WY supervised the data collection carried out by research assistants for all the studies conducted in the marketing behavioral lab at the Nanjing University and Shanghai University of Finance and Economics from Autumn 2016 to Summer 2017 and wrote the manuscript together. All authors contributed to the article and approved the submitted version.

## Conflict of Interest

The authors declare that the research was conducted in the absence of any commercial or financial relationships that could be construed as a potential conflict of interest.
